# Notes on the American Dental Association, 1875

**Published:** 1876-05

**Authors:** S. P. Cutler

**Affiliations:** Memphis, Tenn.


AMERICAN JOURNAL

OF

DENTAL SCIENCE.

Vol. X. THIRD SERIES—MAY, 1876. No. 1.

### ARTICLE I.

### *Notes on the American Dental Association*, 1875.

[*For the American Journal of Dental Science.*]

BY S. P. CUTLER, M. D., D. D. S., MEMPHIS, TENN.

Third day, morning session, *Dental Cosmos*, November, 1875, page 580, subject—Dental Chemistry. Writer says: "The report first alluded to a subject which had been investigated the past year by Dr. S. B. P., a member of the committee, viz: Chemical and Galvanic Action upon Teeth and the Materials used in Filling; Dr. P. assumed that many cases of failure after careful filling are due to electro chemical decomposition of the tooth bone, induced by the current set up between the tooth-bone and the filling material."

This current set up between filling and tooth-bone, is to me high assumption, unless sufficient proof from experiment or rational theory to sustain the statement is given. Now if the tooth be a live tooth, (though no distinction is made,) if there be any galvanic action caused by the filling material, the current would of necessity be conducted by the sensorial nerve fibrils of the tubuli from the point of disturbance, (if any,) to the brain, and a sensation felt in the tooth about the filling; but if the tooth filled be a dead one, no such current could be established. In the former instance, (admitting the fact,) this magnetic current must be a vital nerve current of electro animal magnetism established by the disturbing agency of the filling material, a physical agent, hence so far abnormal, and not a normal or healthful current.

In this case, there must be suffieient chemical action going on adjacent to the filling to establish the current, which must be considerable enough in fact to speedily destroy the tooth, it seems to me much sooner than is actually the case, and the current would be the effect and not the cause of such action; the current certainly could not precede any and all such action; as magnetic action is an effect of a disturbing cause, either chemical action or physical disturbance, such as, motion, impact, friction, collision, changes of temperatures, &c.

Changes of temperature suddenly in the mouth, where there is a very large filling, say gold, where there is much sensibility to such changes, may cause a sudden current of disturbance so as to slightly or severely shock the teeth, and this shock quite independent of all chemical change or action. Such cases come under our observation very often; this is always regarded as unfavorable; in some cases this effect passes away after a few days without any subsequent injury to the tooth, in other cases decay or softening sits in after a time, and a failure is the consequence This softening may be owing both to chemical and galvanic action combined, and the galvanic may aid the chemical or may in fact, start such chemical action, and both together soon works destruction, either by polarising out the atoms of lime by magnetic currents, or decomposed by chemical change, the work of an acid action.

This condition of things often takes place in naturally sensitive teeth when the filling is large in proportion to size of tooth, such as often happens in badly decayed front teeth exposed to sudden thermal changes. Such a condition of things just referred to, cannot exist when a tooth has lost its nerve pulp; although the physical conditions may be intact, the vital conditions are wanting.

If decay follows filling in dead teeth, they are not influenced by neural currents to the brain, and must be confined to the tooth alone, hence the character of decay must be different, in fact, not so rapid or destructive. Precisely how this magnetic vital current affects the character of decay is not quite clear to my understanding.

In other cases where there is no thermal sensibility, decay sets in and runs its course ; in such cases the galvanic influence is not quite so apparent, and if occurring must be in more normal relations to nerve transmissibility, or in fact such currents may be regarded as normal whenever sensitive ; the currents must be pathological.

A line tooth with decay in it is in an abnormal condition, also when filled with any kind of material, more so with some than other materials. In dead teeth, neither decay nor filling has anything to do with its vital connections, and the decay is no cause of disturbance filled or unfilled,—neither can I see how any magnetic action or galvanic, can be established in a dead tooth, from one filling to an other in the same tooth, even if one be made of gold and the other amalgam, as the dead tooth is in all respects a non-conductor of both heat and magnetism.

The writer speaks of latent electricity contained in all matter, as liable to be disturbed under favorable conditions, (as certain fluids,) and currents established and this is an effort to establish equilibrium, will result in a loss of both form and texture in one of the elements, and changing one of the elements of a battery to a higher order changes the polarity. I do not comprehend precisely the application of this statement to the present case in decay of teeth.

He evidently regards the dentine or tooth bone as one of the elements of the battery, while the filling is the other element, hence a falling of one material may be a lower element in the battery, while some other material may be a higher element in the battery, and the polarities will be different; says also, that alkali substituted for the acid in a battery changes the polarity. In this last my experience teaches me that the alkali would stop all magnetic action, by non-action on the zinc or anything else used.

The filled tooth and the fluids of the mouth constitute the battery under consideration, and a decay may be set up when there is an acid condition, and when there is an alkaline condition or neutral there should be no decay, as there will be no chemical action and no galvanic action follows.

When a gold filling occupies one side of a tooth, and an amalgam filling on other side of the tooth, (according to one statement made further on in the transactions,) these two fillings now constitute two elements of the battery, and a decay will take place about the gold filling; quoting from Dr. W.—"A thunder mill was established in the mouth," a battle of the elements it must be he means—page 583.

In this case tooth bone is not regarded as an element in the battery at all; how is this? is this an exception? Further says, "In an abnormal or porous condition of tooth bone, gold by reason of its negatives properties, supplies a decomposing current, while the lower grades of filling material being more nearly on the same plane of equilibrium with dentine, less action is produced and less breaking down of tooth structure. Tin not only arrests decay mechanically, but in frail chalky structures acts as an out acid element, arresting the current. Gold is best in well organized teeth. Amalgam stands higher in the scale, and like gold is liable to excite chemical action. Says, if there is not perfect combination, the mercury will be acted upon by the current induced by the finer element, and the effect is visible in the fine pits which we see upon the surface and edges of the plug. Any attempt to make the amalgam finer is a step in the wrong direction."

How it is that gold can establish a decomposing current in frail teeth and not in sounder structures is not so clear, or how any current at all can be established by the gold independent of any chemical action is not so clear, unless we account for it on that of thermal changes. Neither is it quite so clear how gold can induce chemical action when it suffers no chemical change, while tin is regarded as more saving of frail teeth, when it is admitted that it does oxidize or undergoes chemical change. How tin acts as an anti-acid is not stated; we are to infer that tin in some way keeps up an alkaline influence, at least around the filling; if true, tin is certainly the best thing to use even in all cases.

Speaking of amalgams, says, if there is not perfect combination the mercury will be acted upon, and here we are to infer that the mercury alone is acted on which causes the pitting and ragged edges. Right here let us demur; why? let us see. When the zinc in a galvanic battery is amalgamated with quicksilver, the sulphuric acid goes through the coating of mercury and acts on the zinc without acting on the mercury at all, comparatively, while the zinc is consumed, forming sulphate of the oxide of zinc; it certainly cannot be the mercury that forms the dark color *oxide;* when mercury oxidizes, the oxide is red, not black or dark at all; that of silver is dark; that of tin is white, used in polishing as putty powder, hence the dark color in amalgams comes from the silver *argenture.*

Page 582, says "Gold and tooth bone form a battery, proven by experiment, then says of tooth bone, in firm teeth it is a non-conductor, but in soft porous teeth it becomes a conductor, and the fillings become loosened. We can measure this action with the galvanometer; says tin possesses antiseptic properties which gold does not possess."

Does the speaker mean that a battery can be formed by gold and tooth bone and saliva, or some other liquid as an acid both in or out of the mouth so as to influence the galvanometer sensibly? If so, how can the test be applied in the mouth? has this been done at all practically or only theoretically? But he leads us to infer that such a battery can only be formed where the teeth are soft and porous. Here nature must make great discrimination as to the quality of material. Such a rule certainly does not apply to zinc, whether of good or bad quality used in a battery, so that the acid will act on it at all.

I would ask in what way tin in its metallic state can possess antiseptic properties any more than gold or any other metal as such? If such be the case, it must depend more on its non-conducting properties than anything else. How boiling a gold plate in alkali, (strong soda,) can change the surface, (as spoken of on same page,) is not very clear, only so far as cleaning the plate is concerned.

Same page, Prof. S. inquired why it was that when gold and amalgam are in close contact, that the dentine is apt to be destroyed around gold? Dr. P. said that it was because there was an alkaline current about the amalgam, but around the gold there was acidity. Has this statement been actually demonstrated in the mouth, or only theoretically?

At all events in this case, the amalgam has the best of it, and saves where gold destroys. Why then should not amalgam be able to save when used by itself? Page 583—All the above experiments are instructive and interesting and in the right direction, and ought to be carried out to their fullest extent and made practical as far as possible; too much light cannot be had on any subject pertaining to diseases, prevention and preservation of the dental organs, and it is from such investigations, experimentation, that a better understanding of all that pertains to the diseases of these organs is to be arrived at. Much more might be said on the subject but space will not admit.

